# Editorial: Green and Sustainable Solutions for Fractionating Lignocellulosic Biomass

**DOI:** 10.3389/fchem.2021.803431

**Published:** 2021-12-03

**Authors:** Daniela Millan, Uroš Novak, André M. Da Costa Lopes

**Affiliations:** ^1^ Universidad Bernardo O'Higgins, Santiago, Chile; ^2^ National Institute of Chemistry, Ljubljana, Slovenia; ^3^ CICECO, Aveiro Institute of Materials, Department of Chemistry, University of Aveiro, Aveiro, Portugal; ^4^ CECOLAB-Collaborative Laboratory Towards Circular Economy, R. Nossa Senhora da Conceição, Oliveira do Hospital, Portugal

**Keywords:** lignin, lignocellulosic biomass, green solvent, sustainability, pretreatment

The use of lignocellulosic biomass as feedstock towards the production of goods and commodities, including energy, fuels, materials, and chemicals is of utmost importance to cover the so desired switch from a fossil-based economy to a circular bioeconomy. The application of the biorefinery concept and green chemistry principles are expected to upgrade biomass valorization activities without compromising the current and future environmental regulations and to provide a more sustainable layout of development. In this sense, research efforts must be placed in the right direction towards sustainability. This topic research addresses some particular methods to achieve green and sustainable fractionation of lignocellulosic biomass, including the development of innovative and selective biomass delignification processes for the valorization of both polysaccharide and lignin fractions, the screening of content variation of biomass components, and last and not least the evaluation of different techniques to measure the catalytic activity of enzymes for biomass polysaccharide depolymerization. In line with the demand for sustainability and the aim of this topic, (Pérez et al.) have proposed a new alternative for delignification in chemical pulping. This methodology combines low-energy mechanical pulp and a deep eutectic solvent (DES) based on lactic acid and choline chloride for wood delignification to obtain fibers with low lignin content. The use of low-energy mechanical pulping for the production of pulp in the paper industry aims at avoiding the energy intensive thermomechanical pulping (more than 2.0 MWh/ton). A cooking process of 1 h at 100°C and a liquor-to-wood ratio of 27:1 was found as best conditions, enabling to achieve a pulp yield of about 65% and a delignification degree lower than 16%. Finally, the authors introduce a new parameter Q, which allows evaluating the impact of the operational conditions to quantify the quality of the pulp in terms of delignification degree and fiber length. Values of Q near to Qmin indicate a low delignification degree and a low fiber length. Correspondingly, values of Q near to Qmax imply a high delignification degree and a high fiber length. This novel parameter can be used with other experimental conditions in the delignification of wood fibers.

The efficient fractionation of lignocellulosic biomass is one of the main topics of research in lignin valorization, for example, the work of (Khongchamnan et al.) proposes an initiative to efficiently extract lignin from the corn stover using a single-step solvothermal fractionation in the presence of H_2_SO_4_ in catalytic amount. The researchers used a mixture of ethyl acetate, ethanol, and water at a ratio of 30:25:45 (v/v) respectively as the reaction media. A solvent mixture that has not been used before to extract lignin from corn stover. This methodology, benchmarked with previous work reported in similar experimental conditions, enhances lignin removal from the solid phase (75%) with a purity of 89%.

The quality and purity of extracted lignin seem to be crucial factors. Regarding this concern, (Zijlstra et al.) investigated the potential of butanosolv extraction in a flow-through set up. They performed a convenient work-up procedure to obtain butanosolv lignin in high yield with high quality (high *β*-O-4 content) and purity (limited carbohydrate impurities). To achieve this, an acidic 9:1 vol% n-butanol/water mixture (0.18 M H_2_SO_4_) was used to extract lignin from walnut shells at experimental conditions of 120°C and 2.5 h extraction time. The remarkable results show a 95% delignification and 85% lignin recovery from the original biomass. Isolated lignin contained impurities of about 12% which can be removed by a more careful washing or adding a cosolvent.

Within the special issue, the research from (Xu et al.) provided an analysis of the natural variation of lignocellulosic components from miscanthus biomass in China. The relationship between the changes in miscanthus plant holocellulose and hemicellulose in respect to the geographical growth locations was determined for the 179 miscanthus accessions via acid hydrolysis and high-performance liquid chromatography showing an enriched genetic diversity. The distribution of lignocellulosic biomass is presented as an important factor affecting both the conversion efficiency of biomass energy plants as well.

Finally, to address a complete bibliographic research, (Wang et al.) provided an updated review of recent advances in screening methods for the functional investigation of lytic polysaccharide monooxygenases (LPMO). These newly discovered and widely studied enzymes play a key role in the depolymerisation of sugar-based biopolymers (including cellulose, hemicellulose, chitin, and starch), and exhibit a positive impact on biomass conversion. The review assessed the various LPMO activity analysis methods reported so far, including mature mass spectrometry, chromatography, labeling, and indirect measurements, and summarized their advantages, disadvantages, and applicability.

In conclusion, this special issue demonstrates some advances and new technologies related to the fractionation and conversion of lignocellulosic biomass, helping to build a path to a more sustainable valorization of this kind of renewable materials.

